# “A debriefer must be neutral” and other debriefing myths: a systemic inquiry-based qualitative study of taken-for-granted beliefs about clinical post-event debriefing

**DOI:** 10.1186/s41077-021-00161-5

**Published:** 2021-03-04

**Authors:** Julia Carolin Seelandt, Katie Walker, Michaela Kolbe

**Affiliations:** 1 Simulation Center, University Hospital Zurich, Rämistrasse 100, 8091 Zürich, Switzerland; 2 New York City, Health + Hospitals Simulation Center, 1400 Pelham Parkway South, Building 4, 2nd Floor, Bronx, NY 10461 USA

**Keywords:** Debriefing, Qualitative research, Implicit theories

## Abstract

**Background:**

The goal of this study was to identify taken-for-granted beliefs and assumptions about use, costs, and facilitation of post-event debriefing. These myths prevent the ubiquitous uptake of post-event debriefing in clinical units, and therefore the identification of process, teamwork, and latent safety threats that lead to medical error. By naming these false barriers and assumptions, the authors believe that clinical event debriefing can be implemented more broadly.

**Methods:**

We interviewed an international sample of 37 clinicians, educators, scholars, researchers, and healthcare administrators from hospitals, universities, and healthcare organizations in Western Europe and the USA, who had a broad range of debriefing experience. We adopted a systemic-constructivist approach that aimed at exploring in-depth assumptions about debriefing beyond obvious constraints such as time and logistics and focused on interpersonal relationships within organizations. Using circular questions, we intended to uncover new and tacit knowledge about barriers and facilitators of regular clinical debriefings. All interviews were transcribed and analyzed following a comprehensive process of inductive open coding.

**Results:**

In total, 1508.62 min of interviews (25 h, 9 min, and 2 s) were analyzed, and 1591 answers were categorized. Many implicit debriefing theories reflected current scientific evidence, particularly with respect to debriefing value and topics, the complexity and difficulty of facilitation, the importance of structuring the debriefing and engaging in reflective practice to advance debriefing skills. We also identified four debriefing myths which may prevent post-event debriefing from being implemented in clinical units.

**Conclusion:**

The debriefing myths include (1) debriefing only when disaster strikes, (2) debriefing is a luxury, (3) senior clinicians should determine debriefing content, and (4) debriefers must be neutral and nonjudgmental. These myths offer valuable insights into why current debriefing practices are ad hoc and not embedded into daily unit practices. They may help ignite a renewed momentum into the implementation of post-event debriefing in clinical settings.

**Supplementary Information:**

The online version contains supplementary material available at 10.1186/s41077-021-00161-5.

## Introduction

Post-event debriefing is held in clinical settings among healthcare providers and is an educational, team learning, and patient safety intervention [1–4]. It is a guided learning conversation among participants that aims to explore and understand the relationships among events, actions, thought and feeling processes, and performance outcomes of a clinical situation [5–10]. Debriefing, also labelled after-action review [11, 12], is based on mutual reflection of clinical practice. Although debriefing is a core part of simulation-based training and clinical rehearsal, its use in learning from real events in the clinical environment is well documented [10, 13]. It is a low-cost learning opportunity designed for multiple forms of healthcare teams [1, 2, 14, 15], which makes it particularly suited for managing effective and safe teamwork during uncertain, complex, and risky conditions, such as the COVID-19 pandemic [16, 17].

Empirical studies have demonstrated the effectiveness of learning-oriented debriefing [8, 14, 18–20]. Yet in spite of the obvious potential benefits, debriefing is still underused in the clinical environment [1, 21–24]. One contributing factor is the perceived difficulty of facilitating debriefing conversations [22, 25–29]. Although debriefing is best facilitated by trained debriefers, there are literature, courses, and videos freely available on the numerous approaches for how to structure debriefing, create a psychologically safe and engaging setting, use of co-debriefing, and the management of difficult debriefing situations [5, 23, 30–45]. Another contributing factor is logistical barriers such as high workload, interprofessional scheduling issues, social distancing, or lack of interest [22, 23, 46]. These logistical barriers can be addressed through the judicious scheduling of post-event debriefings. Even using no-go criteria, which are standard best practice for in situ simulation, may be useful [47].

Organization science may point to another set of barriers—and enablers—of debriefing: taken-for-granted beliefs about use, costs, and benefits of debriefing. Also called lay or implicit theories, these taken-for-granted beliefs allow individuals to make priori predictions, comparable to scientific theories [48]. Yet, in contrast to scientific theories, “lay theories need not be objective, testable, or true. Lay theories may be adopted to serve the self and to justify the state of affairs” (p. 18) [49]. They reflect organizational paradoxes such as hospital management directing hospital staff to conduct debriefings while simultaneously triggering a culture of anxiety, time pressure, and control, thus, preventing open communication [50]. As such, implicit debriefing theories may include myths which prevent healthcare personnel from engaging in debriefings. This is problematic because it woefully impedes benevolent initiatives to install regular team debriefings as low-cost learning opportunities.

The goal of this study was to identify implicit debriefing theories with a particular focus on debriefing myths—beliefs and misconceptions contradicting empirical evidence. Myths and implicit theories have important functions such as understanding, simplification, and self- and group-protection. They also determine individuals’ and team’s actions [49]. We interviewed an international sample of clinicians, educators, scholars, healthcare administrators, and researchers who had a broad range of debriefing expertise, and adopted a systemic-constructivist approach that aimed at exploring in-depth assumptions about debriefing beyond obvious constraints such as time and logistics and focused on interpersonal relationships within organizations. In particular, using circular questions, we intended to uncover new and tacit knowledge about barriers and facilitators of conducting regular clinical debriefings. Circular questions are based on social constructivism and on circular assumptions about an issue; they aim at exploring recurrent patterns and processes, generating information, fostering perspective taking, “fluidizing” problems, and putting actions into relational contexts [51–57]. They explore interactions with respect to differences in behavior rather than personality traits, ranking and classification, change in the relationship before and after an event, and differences in respect to hypothetical conditions [44, 55, 57].

In a variety of instances, debriefings are the only opportunity for learning. For example, during the COVID-19 pandemic, additional healthcare providers with various levels of training joined intensive care personnel to care for very sick patients in healthcare facilities globally. Teams had to form and function on the spot with little time for formal training. Post-event debriefings held at the end of each shift or if feasible, during the shift as well, can support such ad hoc teams during the pandemic and allow for lessons learned and process improvement, before members disband and join other teams the following day [17, 58, 59]. In this study, we analyze implicit theories of post-event debriefings. Post-event debriefings are defined as any structured post-event discussion with the purpose of learning; their labels may vary across professions and organizations [11, 60]. Importantly, these debriefings are not to be confused with stress debriefings as psychological intervention to prevent or treat traumatic experiences [61, 62]. Addressing the gaps in our current understanding of what facilitates effective debriefing is important for developing and targeting debriefing faculty development efforts for clinical faculty [63–65]. It will also contribute to implementing and maintaining a culture of debriefing and open conversations in healthcare organizations. Actionable knowledge on how to achieve in-depth reflection in debriefing may help mitigate cognitive load during debriefing [28], enhance debriefing skills and debriefing quality, and thus contribute to safer patient care. When the learnings from clinical debriefings have a clear pathway to be actioned, visible quality improvement follows, which may in turn inspire clinicians to debrief again to see yet more improvement.

## Method

This study was ruled exempt by the local ethics committee.

### Setting

Interviews were conducted within the scope of a research project investigating how structured debriefings can provide a suitable learning infrastructure for acute care teams in healthcare. Interviews were conducted by the first and last authors (JS and MK) with backgrounds in organizational psychology and simulation-based team training. All experts participated voluntarily in this study. Interviews took place during regular working hours and were usually conducted at the participants’ workplace. Participants did not receive any credits or benefits for their participation. In total, 1508.62 min (25 h, 9min, 2 s) of interviews were recorded.

### Participants

Of the 37 participants, 10 (27%) were nurses, 17 (46%) physicians, 6 (16%) scholars, and 4 (11%) education specialists and administrators from hospitals, universities, and healthcare organizations in Switzerland, Germany, and the USA. We deliberately approached experts who differed in both the quality and quantity of their debriefing experience. Some clinicians among our study participants had a shared experience with debriefings in both clinical and simulation-based training settings. Other participants had—by the very nature of their occupation as administrators or scholars—not participated in numerous debriefings themselves but had, through research, counselling, or implementation, developed valuable and unique expertise on debriefings. We intended the sample to represent the diversity of healthcare professions who have reflected on how to initiate, conduct, and implement post-event and learning-oriented debriefings. These participants had varying and overlapping degrees of experience from multiple perspectives with it. As such, our recruiting strategy was threefold: First, we recruited clinicians based on our knowledge that they had valuable experience in conducting, participating in, or observing post-event debriefings in hospitals. Second, we recruited interview participants (clinical and non-clinical) who were renowned experts in the fields of team learning, team debriefing and/or team reflexivity—they had not necessarily been frequently participating in debriefings themselves. Third, we included healthcare administrators; some of them had previous careers as clinicians. This recruiting strategy allowed us to have a broad and multi-pronged perspective.

### Data collection instrument

For the purpose of this study, a semi-structured interview guide with questions related to challenges and success criteria for debriefings in the clinical setting was designed. The data collection instrument was developed based on research in organizational behavior (with a particular focus on difficult conversations) [66–70], debriefings in healthcare [5, 26, 30, 38, 40, 63–65, 71–75], and circular questioning [44, 55]. Content areas were (1) experiences with debriefings in clinical and training settings; (2) characteristics of debriefings with respect to participants, place, duration, frequency, and organizational routines; (3) mental models with respect to effectiveness of debriefings and differences between debriefings and other kinds of conversations; (4) leadership in debriefings; (5) psychological safety; and (6) double-loop learning. Interviews were structured along these content areas. Targeted question types were used. For example, for assessing participants’ homogeneity vs. heterogeneity with respect to certain attitudes, scaling questions, a type of question derived from sociometry [76, 77] prompting interviewees to quantify their experience (e.g., “On a scale from 1 to 10, how high do you consider the extent of discussing “unpopular topics” in debriefings – 1 = only popular topics discussed; 10 = only unpopular topics discussed?”), were used. For exploring statements in more detail, a variety of circular questions (i.e., questions based on circular assumptions about relationships and interactions [44, 55]), were applied (e.g., “You mentioned earlier that debriefings are typically run by XY. How do you explain that?”). For desired yes/no answers, close-ended questions were used (e.g., “Do you think there should be rules for how to conduct debriefings?”); for desired narrative answers, open-ended questions were used (e.g., “Which rules do you think there should be?”).

### Data collection

At the beginning of each interview, we thanked the participants for taking time for the interview and informed them about the study objectives and potential interview length of 30 min. One interview was conducted in English; the other interviews were conducted in German. The primary language of most participants was German. Consent for recording and transcription was obtained verbally. All participants were informed that the authors would not publish any answers allowing for personal details of the interviewees. As we conducted interviews rather than conversations, we previewed that we would not engage in an in-depth discussion and would not comment on participants’ statements. We invited the participants to share anything beyond what we were addressing in our questions that they would consider relevant. We also asked for permission to record the interview. The recorded interviews did not contain any personal information about the participants. Data collection was anonymous and confidential. Assignment of participants’ names to their interview data was not possible.

### Data analysis

Formation of categories was done using an inductive open coding process structured along the interview questions (Table 1) [80]. Depending on the selected data analysis method, answers can be excluded (e.g., arguments that do not fit the topic can be excluded and would thus not be analyzed). We decided to use all answers (*N* = 1591) and gradually formed data categories related to our assumptions and the concepts of the relevant literature. Answers were sorted step by step into the categories, and if they did not fit into existing categories, new categories were developed inductively. After having processed a portion of the data, the chosen categories were reviewed. It was examined whether redundant or overlapping categories exist. After reviewing the coding scheme, the final version was established and a coding manual was written before answers were sorted into the final categories, and relative frequencies for each category were determined. Although all participants were asked the full set of questions, they were free to not answer some of the questions. Some questions were only answered by three participants. Due to the large amount of data, we decided that questions needed to be answered by at least one third of the participants (*n* = 12) to be categorized. As a final step, debriefing myths across categories were extracted.

**Table 1:** Data analysis process

Step	Procedure
1	A master’s student holding a bachelor’s degree in psychology transcribed all 25 h, 9 min, and 2 s of recorded interviews.
2	Another master’s student holding a bachelor’s degree in psychology arranged all transcribed interviews in an Excel spreadsheet for more feasible analyses of content. This Excel spreadsheet did not contain any personal information about the participants.
3	JS and MK reviewed transcribed data and generated a list of rough categories in an open-coding process.
4	JS and MK reviewed rough categories and identified clusters of categories, which they discussed and revised. A preliminary coding list resulted with categories describing the themes of the debriefing myths and containing a description of their contents.
5	While using an iterative process [27], JS and MK moved back and forth between the original transcribed data, their assumptions, concepts of the relevant literature (e.g., single-loop / double-loop learning [70], psychological safety [69]), and the emerging categories. After having processed a portion of the data, the chosen categories were reviewed, and it was examined whether redundant or overlapping categories exist.
6	The final version of the coding scheme and the categories, respectively, were then applied by JS and MK for re-coding the complete data set.
7	For ensuring inter-rater reliability, JS and MK independently coded 15% (238/1591) of the material [78]. Cohen’s Kappa was .91, indicating very good inter-rater reliability [79].
8	Absolute and relative frequencies for all categories were determined.
9	Extracting debriefing myths, i.e., beliefs in contrast to existing scientific evidence

## Results

As expected, reported practical debriefing experiences varied: Twenty-seven participants reported only some practical experience with post-event debriefings (i.e., conducting or participating in). These participants shared their views from a clinical (i.e., many information-sharing meetings, but no explicitly established debriefing routine yet), scholarly (i.e., studying team reflexivity) and/or administrative (e.g., overseeing patient safety initiatives) perspective. Others reported significant experience with more than 100 debriefings (see Supplementary Table 1, Additional file 1).

In what follows, we first briefly report findings on debriefing characteristics and beliefs about debriefing value; results of the respective coding are included in the supplemental digital content (see Supplementary Table 2, Additional file 2 & Supplementary Table 3, Additional file 3). Second, we focus on findings with respect to beliefs about what to talk about and taboo topics in debriefing (Tables 2 and 3). Third, we describe reported beliefs about debriefing facilitation (Table 4 and Supplementary Table 4, Additional file 4) and about learning environments (see Supplementary Table 5, Additional file 5). The percentage of each answer is indicated in parentheses. We discuss implicit debriefing theories that qualify as debriefing myths in the subsequent discussion section.

**Table 2:** Beliefs about debriefing topics and who gets to decide on them

Key themes	Representative quote	%
***Based on your observations, what do people typically talk about during debriefings?***		
Technical and medical issues	“Mainly technical issues [...] I really have to make an effort not to stick to technical stuff.”	30.2
Teamwork	“[...] one of the classic issues is collaboration among the anaesthetists and the trauma team in the trauma.”	20.8
Critical events and mistakes	“Mostly about mistakes.”	18.9
Room for improvement	“Room for improvement is a major topic in our department.”	15.1
Emotions	“[…] the emotional, the mental component, how was I feeling, what issue does she have, these aren’t always just constructive, because they are exaggerated and very much dramatized.”	9.4
Reflection	“ […] they reflect about the situation, why he or she acted this way, what his or her considerations were […].”	5.7
***What should be talked about in debriefings?***		
Teamwork	“[…] about working together, this is swept under the table.”	29.2
Emotions and perceptions	“I do believe that there should be room for feelings or good feelings in debriefings.”	18.5
Critical events and mistakes	“As unpleasant as is it, (but) we should try to talk about the things that didn’t go well [...].”	13.9
Technical and medical issues	“In the daily clinical routine, the focus is on technique, you have to show and teach your people the basic tools.”	13.9
Room for improvement	“[…] what were the reasons that something didn’t go as planned, such things, yes.”	12.3
Reflection	“[…] but also what was going on in the people’s minds, how they understood it [...].”	6.2
Uncertainty	“I believe we should talk about things that […] entail uncertainties.”	3.1
The complete case	“About the whole procedure, about all scenes, from the beginning until the end, because in the very situation so many things are happening that are running through your mind.”	3.1
***Who do you think has the most influence on what is talked about in debriefings?***		
Debriefer and initiator	“[…] the instructor […] he or she should conduct the conversation.	31.2
Senior / more experienced staff members	“Basically, I would say that experienced staff have more influence, because they feel more confident in their roles.”	27.9
Physicians	“There are indeed attendings who determine what is talked about.”	21.3
Personality	“In principle, I believe that extroverted people say a lot more in a debriefing that those who are reserved, which you can’t change in a debriefing.”	14.7
Everybody	“I believe that everyone has influence, everyone who dares to.”	3.3
Culture	“It very much depends on the culture of the [...] groups.”	1.6
***Who do you think has the least influence on what is talked about in debriefings?***		
People with certain personality characteristics	“Introverted staff will be rather quiet […]”	42.9
Less powerful staff members	“The least the lowest-ranking […]”	20
Nurses	“The nurses are the most reserved.”	14.3
Those with little involvement	“[…] the least the ones who are hardly involved […]”	8.6
Residents	“What I observe is that we, the attendings, do have the most influence on what is talked about because […] the residents keep listening to us automatically.”	5.7
It depends on the respective person	“I believe it depends very much on the person and on whom you’re talking to.”	5.7
Nobody	“… I don’t believe that there is someone who doesn’t have any influence.”	2.9
***How do you explain that?***		
Impact of hierarchy	“The highest-ranking person makes the plan and the rest follows him or her blindly.	26.5
Impact of facilitation	“…the moderator conducts the discussion, but everyone should be able to have a say in it.”	26.5
Impact of experience & expertise	“[…] but they must have some idea of the topic”	20.4
Impact of role & position	“This depends entirely on the role of a participant […]”	12.2
Impact of personality characteristics	“… on the one hand with the […] power/assertiveness of the initiators.”	6.1
Impact of logistics	“This has something to do with the varying shifts and handovers, this makes it difficult […]”	8.2
***In your view, who should have the most influence on what is talked about in debriefings?***		
Senior / more experienced staff members	“I think it is the hierarchical structures in medicine, the person at the top has most influence, and the rest just obeys.”	40
Debriefer	“The person who facilitates the conversation has most influence. They should create an atmosphere in which employees feel comfortable and free to speak up. […]”	36.7
Everybody	“[…] if anyone can initiate it as they please, then there should be no differences.”	15
Other	[…] not so much related to the person […] and then not the person with the highest status or the loudest person, […] the most crucial aspects should come up for discussion.	8.3

**Table 3:** Beliefs about taboo topics in debriefing

Key themes	Representative quote	%
***If there were less popular topics in debriefings, maybe even taboo topics, which would that be?***		
Mistakes, errors, and deviations	“If someone had made a mistake, […], I only noticed this once, they pretended it had never happened, they made minimal corrections, and then they pretended it had never happened […].”	32.9
Hierarchy	“[…] Addressing problems related to working conditions and superiors, anything that could challenge hierarchy […]”	20.6
Personal issues	“[…] I find it extremely difficult to talk on a personal level. Addressing personal issues or criticizing someone […]”	19.2
Sexual harassment	“[…] Topics related to sex, harassment and other delicate issues […]”	9.6
Emotions	“Most often it is rather emotional topics that make someone feel offended or when is situation itself is a demanding.”	6.7
Cultural differences	“[…] Teamwork between nurses and physicians is always a hot topic […]”	5.5
Nothing	“The stronger the trust within the team, and in the attending physician, there are no taboo issues that can’t be talked about. In my department, I know of nothing that can’t be talked about […]”	2.7
Ethical issues	“[…] extreme situations, deciding when resuscitation efforts should be stopped or how relatives should be involved […]”	2.7
***On a scale from 1 to 10, to what extent do you think people talk about unpopular topics in debriefings; 1 = only talk about popular topics, 10 = only talk about unpopular topics?***		
3–4		34.4
7–8		31.3
5–6		25
1–2		9.4
***On a scale from 1 to 10, are these unpopular topics typically addressed at the beginning or at the end of debriefings; 1 = at the very beginning, 10 = at the very end?***		
7–8		47.6
5–6		23.8
1–2		19.1
3–4		9.5
***In your view, who has the best ability to address unpopular issues?***		
External person	“I would say an external person […], people who are not involved in but still somewhat knows the process. […]. Someone standing on the side lines still knowing what is going on.”	25
Staff members who are trained and experienced	“Someone with appropriate training and experience.”	19.6
Staff members who are open minded	“[…] I guess just being open and giving anyone a voice.”	17.9
Staff members who are popular and brave	“[…] an empathic person […] who is not afraid of repercussions [...] who will not blush and backtrack [...]”	12.5
Senior / more experienced staff members	“There is a clear hierarchy. The people on the highest hierarchical level have better opportunities to address something […]”	10.7
Involved team members	“Well, I think it would be best if someone within the team would run it. In my opinion, it is easier that self-awareness arise within the conversation instead of discussing it from my point of view as a leader.”	5.4
Physicians	“Since conversations are mostly or frequently conducted by physicians, they have the greatest chance to address issues, treat them transparently and motivate the nurses.”	5.4
Psychologists	“[…] Probably psychologists, they are open for debriefings and have therefore the best requirements […]”	3.6
***In your view, who has the least ability to address unpopular issues?***		
Staff members without debriefings experience	“Someone without appropriate training.”	27.6
Staff members who are unpopular	“The bullying head of department.”	20.7
Staff members who cannot listen	“People who cannot listen.”	13.8
Junior staff members	“[… ] lower hierarchy level would have fewer chances.”	13.8
Physicians	“Physicians because they do not wish to speak about mistakes, especially older physicians.”	6.9
Involved team members	“[… ] those who are the most affected [… ].”	6.9
Nurses	“[… ] the least requirements have nurses.”	6.9
People taking care of patient positioning	“If people taking care of patient positioning would address unpopular issues, everyone would be like ‘oh no, we do not have to listen to them’.”	3.5

**Table 4:** Beliefs about facilitating debriefing

Key theme	Representative quote	%
***If you could change the way debriefings are typically led, what would you do?***		
Define who facilitates	“I would have the person with the lowest rank run it, because it would turn the hierarchy upside down, symbolically and in the debriefing, and there would be more input as if an attending were running it.”	65.4
Institutionalize debriefing	“[… ] that it will be implemented from above […]”	26.9
Apply a structure	“[…] if at the end there would be another round for questions or further issues [...]”	7.7
***From the perspective of the debriefing participants, what do you think a person who is running the debriefing should do to do a good job?***		
Apply a structure	“[… ] the important structure in this is to give participants room to say what they wish to say, not to intimidate them, and the others should not think that that’s not important.”	28
Be curious	“[… ] must not already have a solution for the problem […]”	16
Be neutral	“He/she must be neutral […]”	13.3
Set the stage and preview	“What I realize again and again is that participants need to know what to expect, a good preview, how it is run, expectations, structure, […]”	13.3
Give space by asking questions and listening	“First of all they need to listen well, make notes. […]”	10.7
Be respectful	“They need to respectfully deal with what the participants say; distribute their attention evenly [… ]”	8
Be competent and have a standing	“To do their job they need to have a certain standing, you cannot give in and be afraid. […]”	6.7
Share point of view	“[…] , I found it really bad why she beats around the bush. I had the feeling that I did not learn enough although she had noticed something […]”	4
***From the perspective of the participants of a debriefing, what do you think the person who is running the debriefing should not do at all?***		
Judge or take sides	“What he or she must not do is take sides or judge […]”	31.5
Be rude or humiliate learners	“[…] nothing condescending […]”	18.5
Assume to hold the truth	“That person must not engage in monologues like ‘that is the debriefing and I will tell you what happened’ […]”	18.5
Blame or abuse power	“[…] no condemning or finger pointing, […]”	18.5
Scare and get scared	“The person must not be daunting, triggering reactance.”	9.3
Laissez-faire	“[…] Then it’s like a slugfest and conflict; the debriefer needs to intervene and must not let it happen. The debriefer should deescalate [...], while at the same time monitor topics that are important to him/her.[…]”	3.7
***In your view, what are the three biggest barriers for the person who is running the debriefing?***		
Lack of debriefing skills	“[…], that you don’t know exactly what and how […]”	26
Fear that participants might not wish to be debriefed	“If somebody disapproves of debriefing, this can be a barrier; to motivate him or her might be a big barrier.”	19.2
Being emotionally involved or not neutral	“Neutrality, if somebody from anaesthesia runs a professional discussion it will be tricky to address the surgeon’s mistake.”	17.8
Logistics	“[…] the temporal aspect, when and how long will it take place and will everybody be there […]”	11
Fear of having to be honest / exposing oneself	“[…] Maybe the skill to criticize somebody […], and that you have to talk to somebody and criticize him/her”	8.2
Fear to speak up / address certain topics	“[…] The topics that you talk about, they quickly get you helpless. You are safe with the medical stuff, but with everything else it gets difficult.”	6.7
Hierarchy	“[…] That you see things too much from your own hierarchical perspective.”	4.1
Repercussions	“You don’t necessarily make yourself popular with addressing certain topics. It can put a strain on the team climate if topics such as misconduct by people get addressed [...] You can be proclaimed as someone who is just robbing you of your time which you don’t have at all after an ten hour shift […]”	4.1
Unreasonable participants	“[...] a lack of understanding from the participants – about what you are talking about or with respect to the error that had happened. […]”	2.7
***On a scale from 1 to 10, how confident do you feel in running a debriefing in clinical practice; 1 = very unconfident, 10 = very confident?***		
5–6		37.5
7–8		31.3
3–4		18.8
1–2		6.3
9–10		6.3
***What do you need to feel even more confident?***		
(Reflective) practice	“Clearly the analysis of it […]”	61.1
Training	“Good training which is comprehensible, simple, easy to remember and feasible, […]”	13.9
Support (e.g., preparation, tools)	“[…] an aid, a checklist or guide […]”	13.9
Observing others debrief	“[...] It helps me to read about it and watch other debriefings.”	11.1

### Characteristics of debriefings with respect to participants, place, duration, frequency, and organizational routines

Reported characteristics of debriefing differed vastly (see Supplementary Table 2, Additional file 2). While the majority described the conduct of various but not classical debriefing types (39.3%; e.g., team meetings, case reviews, etc.), some participants described debriefings following critical events (33.9%), while others talked about the lack of debriefings (14.3%). Frequency (e.g., several times a year (16.1%), rarely (12.9%), daily (12.9%)), duration (e.g., few minutes (22.2%), half an hour (30.6%), three quarters of an hour (5.6%)), participants (e.g., everybody involved (21.6%), physicians and nurses (18.9%), only nurses (18.9%)) differed as well. Who initiated (e.g., attending physician (25%), most senior person (17.5%), nurses (17.5%)) and led the debriefing or meeting (e.g., attending physician (28.6%), most senior person (25%), no one (14.3%)) differed as well with a tendency for seniority. Most debriefings seemed to follow a specific structure (77.3%) as opposed to no structure (18.2%) and take place at various locations (e.g., private setting without disruptions (31.4%), anywhere (14.3%), break room (14.3%)).

### Beliefs about value of debriefing

Participants expressed deliberate value of conducting debriefings for a wide range of people (e.g., participants, 26.8%), including patients and their relatives (24.4%) and staff (19.5%) (see Supplementary Table 3, Additional file 3). Reported debriefing benefits include, among others, higher quality and fewer mistakes (40%), higher work satisfaction (28.9%), and more open communication (20%). Management and staff members with a negative attitude (34.6%) were perceived to benefit the least from debriefings followed by people working in management (15.4%) and people that were not involved (15.4%). Saving time by not conducting debriefings was associated with less learning (38.5%). To support the use of debriefings, culture change (42%), tool availability (32%), and improved logistics (16%) were most frequently mentioned.

### Beliefs about what to talk about and taboo topics in debriefings

Technical and medical issues (30.2%), teamwork (20.8%), and critical events and mistakes (18.9%) were the most frequently mentioned topics that people reported to actually talk about in their debriefings (Table 2). When asked about what should be discussed in debriefing, similar topics were mentioned (teamwork (29.3%), critical events and mistakes (13.9%), with the frequent addition of emotions and perceptions (18.5%)). The exploration of successful performance episodes was not mentioned. Interestingly mistakes, errors, and deviations (32.9%) were also the most frequently mentioned taboo topic for debriefing, closely followed by hierarchy (20.6%) and personal issues (19.2%) (Table 3). Participants seemed divided with respect to the extent that people discuss unpopular topics in debriefings and the respective timing; 47.6% answered that these topics are rather addressed at the end of the debriefing. The best requirements to address potential unpopular topics would have either external people (25%) or trained, experienced (19.6%), or open-minded staff members (17.7%).

The people initiating and facilitating the debriefing (31.2%), senior and more experienced staff members (27.9%) and physicians (21.3%), were described to have (and ought to have) the most influence on what is discussed in debriefings, whereas people with certain personality characteristics (e.g., quiet, introverted members) were described to have the least influence (42.9%), followed by less powerful members (20%) and nurses (14.3%). Participants explained this mostly as the impact of hierarchy (26.5%), facilitation (26.5%), and experience and expertise (20.2%) (Table 2).

### Beliefs about facilitating debriefing

Participants reported a variety of beliefs about facilitating debriefing (Table 4). When asked what would they do if they were able to change how debriefings were typically led, the majority would more clearly define the facilitation role (65.4%). Institutionalizing debriefing by implementing it in a top–down way and making it a deliberate routine was the second most frequent recommendation (26.9%) followed by applying a structure (7.7%). Facilitators should first and foremost apply a structure (28%), be curious (16%) as well as neutral (13.3%), and apply mechanisms for coordinating the conversation, such as setting the stage and previewing (13.3%), giving space by asking questions, and listening (10.7%). Participants reported detailed assumptions about what characterizes a good debriefer like being respectful, standing and staying calm (24.65%), skilled in facilitating conversations (21.5%), and being empathic (13.9%) (see Supplementary Table 4, Additional file 4), as well as actions which she/he should avoid (e.g., judging or taking sides (31.5%), such as being rude or humiliating learners (18.5%), or assuming to hold the truth (18.5%); Table 4). The notion of the debriefer’s “neutrality” and avoidance of judgments occurred frequently. The biggest reported barriers for running a debriefing were a lack of debriefing skills (26%), fear that participants might not wish to be debriefed (19.2%), and being emotionally involved or not neutral (17.8%) (Table 4). To develop more confidence in being able to debrief, most participants reported the need for reflective practice (61.1%) rather than training (13.9%) or support (13.9%; e.g., preparation, tools).

### Beliefs about the learning environment

Participants generally expressed regard for debriefing rules and structure (97%). They seemed hopeful that psychological safety—“the perception of the consequences of taking interpersonal risks” [69]—could be established for debriefing, particularly through providing structure, transparency, managing time, using an appropriate setting and stating objectives (25.4%), providing confidentiality (15.9%), getting support via trained, neutral and authentic debriefers (12.7%), and more (see Supplementary Table 5, Additional file 5). Participants seemed to prefer discussion rather than unilateral feedback in debriefing. However, they also reported that their colleagues would not have much patience in exploring potential reasons for mistakes in detail. The main reported hurdle for exploring mistakes in detail were repercussions (64.3%).

## Discussion

The goal of this study was to identify implicit debriefing theories, i.e., taken-for-granted beliefs about use, costs, and benefits of post-event debriefing. In contrast to scientific theories, implicit theories need neither be objective, nor testable, nor true [49]. They reflect organizational paradoxes [50] and may include myths which prevent healthcare personnel from engaging in debriefings. In what follows, we first summarize the implicit debriefing theories that seem to be in line with recent debriefing science. Second, we highlight debriefing myths, i.e., beliefs and misconceptions that seem to be in contrast to scientific debriefing evidence. Lastly, we point to study limitations.

### Implicit debriefing theories in line with scientific evidence

In line with scientific evidence, the value of debriefings for debriefing participants, patients and their relatives and employees [1, 15, 17, 20] was shared among the study participants—a value that might not be easily visible for management. Participants were also mindful of the delicacy of choosing debriefing topics [67]. Respectively, participants seemed aware of the complexity and difficulty of facilitating debriefings [25], the importance of structuring the debriefing conversation [8, 23, 75] and engaging in reflective practice to advance debriefing skills [26, 27]. The importance of organizational support of debriefing was also shared knowledge [74].

### Debriefing myths

Based on the coded responses presented in detail above, we have identified four debriefing myths. They are presented in Table 5. In what follows, we discuss why each of these myths is problematic.

**Table 5:** Debriefing myths

No	Debriefing myths	Content	Examples
1	Debriefing only when disaster strikes	Belief that particularly negative events or major errors call for debriefings. No reported routines for debriefing successful performance episodes.	“In case of overload, when something went wrong […]” “Mostly after stressful situations […]”
2	Debriefing is a luxury which may not improve team performance.	Belief that debriefings require extra effort that overshadows their benefits: conducting debriefings takes time, and their benefits might not be obvious immediately.	“[…] the temporal aspect, when and how long will it take place and will everybody be there […]” “[…] due to shift work, it is problematic to bring all participants together.” “[…] we do not have time to discuss different points in detail […].”
3	The senior clinician should determine debriefing content.	Experienced and powerful staff members determine what is talked about in debriefings.	“[…] it is structured by hierarchy, I think it is rather the attending physician […]” “Basically, I would say that experienced staff have more influence, because they feel more confident in their roles.” “[…] we attending physicians have most influence on what is talked about because nurses and residents automatically listen to us […]”
4	Debriefers must be neutral.	Debriefers are supposed to be neutral and nonjudgmental.	“[…] it requires a person that is neutral and does not polarize […]” “What he or she must not do is take sides or judge […]” “He/she must be neutral […]”

#### Debriefing myth #1: debrief when disaster strikes

The first debriefing myth we identified through coding describes the assumption that debriefing should particularly—and almost exclusively—follow critical performance episodes and catastrophic events. While many respondents saw the benefit of regular debriefings, the conduct of debriefing seems associated with adverse events, errors, and mistakes—interestingly with mistakes as the most frequently mentioned taboo topic (Tables 3 and 5). This notion seems common; even the recent WHO guidance for after-action review limits its scope to emergencies [12]. This is problematic for several reasons. First, employees may implicitly anchor debriefing with “somethings must have happened” or “I did something wrong” [81]. Since organizational culture tends to consider mistakes as something to avoid, employees may experience fear, anxiety, and embarrassment when asked to debrief and engage in face-saving strategies such as withdrawal, obscuring critique, and reluctance to speak up or to discuss mistakes [26, 68, 82–84]. This process may vastly limit debriefing effectiveness [41]. Second, psychological research has demonstrated that “bad is stronger than good”: bad events and negative information receive far more processing and impact than good events and positive information [85]. As a consequence, debriefing only when disaster strikes may undermine learning from positive performance episodes and overemphasize mistakes, error, and adversity. Third, recent safety approaches highlight the importance of not only exploring why “things go wrong” (i.e., Safety I) but also why “things go right” (i.e., Safety II) [86]. It is recommended not to limit debriefings to learning from failure, and to extent them to learning from success.

#### Debriefing myth #2: debriefing is a luxury which may not improve team performance

The second debriefing myth identified includes the belief that debriefing is something “extra” (Table 5). This myth is not only problematic because it may negatively impact the decision about whether to conduct a debriefing or not when resources are limited. Two factors may contribute to this myth: First, debriefing takes initial extra effort to organize, both explicitly and implicitly [74]. Second, the benefits of debriefings for improved patient care and safety may not seem to be obvious immediately. Importantly, this myth stands in contrast to scientific evidence: almost a decade ago, a meta-analysis showed that learning-oriented debriefings improve performance by 20 to 25% on average [20]—a finding that has been confirmed in a more recent meta-analysis [11]. Team science considers shared reflexivity as occurring in debriefing a core process of teamwork [15, 87–89]. The conduct of debriefing has been associated with more team helping and workload sharing, more speaking up, shorter surgical duration, and reduced number of adverse events [14, 18]. Comparable to the use of checklists, time-outs, and even breaks, debriefing is an investment worth its effort [90–92]. In countries particularly such as the USA, malpractice claims might be reduced due to the impact of debriefing [93, 94]. While the fact that much of today’s patient care being performed by teams and multi-team systems is becoming more and more established [95–98], debriefings may not yet be considered a part of that establishment.

#### Debriefing myth #3: the senior clinician should determine debriefing content

The third debriefing myth describes a dilemma of identifying what to talk about in debriefings; on the one hand, employees of higher seniority and more experience were considered as those who do and should determine debriefing topics. On the other hand, hierarchy was considered a barrier for speaking up with topics during debriefing, especially by quiet and less powerful team members or by professional groups such as nurses. This dilemma is problematic: facilitating is an advanced skill which may not be automatically acquired with more seniority [25]. Leaving the decision of what to talk about with the powerful may manifest undiscussable issues [74]. Research on organizational silence has demonstrated that healthcare professionals may remain silent in spite of having patient safety concerns [99–105]. This silence is caused by a complexity of individual, interpersonal, and organizational barriers to speaking up [99, 106–114]. Clarifying roles and applying predefined debriefing frameworks that suggest relevant content areas such as PEARLS [30], guided team self-correction [19], TeamGAINS [34] may help navigate debriefing content more effectively [115].

#### Debriefing myths #4: debriefers must be neutral and nonjudgmental

The final debriefing myth we have identified includes the belief that debriefing facilitators must be neutral and nonjudgmental. Only three participants (4%) believed that debriefing facilitators should share their point of view. While this myth may reflect the differing perspectives of the participants, it likely reflects the facilitators’ well-described feedback dilemma in debriefing: caring for the personal relationship vs. caring for task performance [26, 116]. They worry that offering critique may damage relationships and make learners defensive, while “protecting” learners by withholding critique may leave them without learning [26]. As a consequence, facilitators are at risk of withholding their personal—positive, negative, astonished, etc.—reactions, leaving the learners in doubt and impeding shared in-depth reflection for the purpose of learning. This might be particularly the case when debriefing colleagues, peers, or even superiors. This myth is problematic for several reasons; first, without honest feedback and curious inquiry, debriefings are shallow, vague, and ineffective [5, 26]. Second, by not offering any personal views, facilitators may manifest “undiscussable” issues and may miss a chance to demonstrate feedback and difficult conversation skills [5, 26]. Third, psychological safety may suffer when facilitators are not “real” [41]. Fourth, critical topics may not be discussed at all [117]. Importantly, group decision-making and counselling science do not suggest to reverse the myth and offer unfiltered judgement, taking sides, or becoming disrespectful. Refraining from neutrality should not be mistaken with arguing about who is right. On the contrary, sharing thoughts, opinions and information in groups is a highly complex process where minor variations in the interaction are associated with major changes in the result [118–121]. Debriefing facilitators are advised to use expertise wisely, at appropriate times and put it up for discussion [7, 60, 66, 121, 122]. By offering one’s point of view at appropriate times and inquiring about others, the facilitator serves as expert mediator, makes the issue “discussable”. Instead of taking sides, facilitators assume multipartiality, even with respect to their own points of view [44]. Since there “is no such thing as nonjudgmental debriefing” [123], holding learners in high regard while assuming curiosity, combining sharing own observations and respective personal reactions with inquiring the learners’ perspective [5, 124] are recommended alternatives. Helpful strategies are establishing a safe learning environment, applying debriefing frameworks, using co-debriefing, getting peer feedback and respective faculty development [40–43, 64, 65, 125].

### Limitations 

This study has limitations. We investigated a comprehensive yet small sample of experts. We deliberately approached experts with varied backgrounds in debriefing experience. Some clinicians among our study participants had a shared experience with debriefings in both clinical and simulation-based training settings. Other participants had—by the very nature of their occupation as administrators or scholars—not participated in numerous debriefings themselves but had, through research, counselling, or implementation, developed valuable and unique expertise on debriefings. Although participants likely represent the diversity of healthcare professions who have reflected on how to initiate, conduct, and implement post-event debriefings with varying and overlapping perspectives, this diversity may include various biases of participants which may warrant further systemic study. Furthermore, participants’ responses were likely triggered by the nature of the interview questions. Since this study deliberately relied on open questions based on organizational behavior science rather than on more narrow vignettes or stimulated recall, we assume that participants might indeed have a broad range of debriefing situations in mind. Also, some participants seemed to easily navigate their narratives with respect to work “as done” and work “as imagined”; however, this differentiation seemed less feasible for others. Data analysis may have been shaped by our own understanding of related phenomena. Further research on debriefing strategies such as embedded in “circle up” [126] may help us understand the efficacy of embedding daily debriefings in clinical units, rather than as an ad hoc tool when disaster strikes. For example, understanding the return on investment (ROI) of debriefing by quantifying the fiscal expense of staff time versus the financial gains of up to 25% improved teamwork, shorter surgical duration, and decreased adverse events might shed even more light on debriefing effectiveness.

## Conclusion

Exploring taken-for-granted beliefs or implicit theories about clinical debriefing can help us understand why powerful quality improvement techniques, such as post-event debriefing, have seen slow uptake in most clinical environments. Despite empirical data demonstrating improved clinical unit performance in several dimensions, for example, less errors and better teamwork; these priori predictions create barriers that prevent clinical leaders endorsing and using debriefing in a routine way. The four debriefing myths highlighted in this manuscript may assist Clinical Leaders and Quality Improvement and Patient Safety personnel to counter false assumptions raised by debriefing skeptics. As simulation educators, we recognize that high-quality debriefing requires a rigorous approach with skilled personnel. We also recognize that the benefits of improved communication and coordination in healthcare teams, and teams that learn and grow together through routine clinical debriefings, far outweigh the costs. We hope that the four presented myths will spark controversy and stimulate more empirical debriefing research.

## Supplementary Information

**Additional file 1: Supplementary Table 1.** Absolute frequencies of study participants experiences with conducting, participating in and observing debriefings.

**Additional file 2: Supplementary Table 2.** Characteristics of debriefings with respect to participants, place, duration, frequency, and organizational routines.

**Additional file 3: Supplementary Table 3.** Beliefs about value of debriefing.

**Additional file 4: Supplementary Table 4.** Characteristics of a “good debriefer”.

**Additional file 5: Supplementary Table 5.** Beliefs about the learning environment of debriefings.

## Data Availability

The datasets used and/or analyzed during the current study are available from the corresponding author on reasonable request.
