# Supplementary material for: “A debriefer must be neutral” and other debriefing myths: a systemic inquiry-based qualitative study of taken-for-granted beliefs about clinical post-event debriefing
Source: Adv Simul (Lond). 2021 Mar 4;6:7. doi: 10.1186/s41077-021-00161-5 (PMC7931165; doi:10.1186/s41077-021-00161-5)
Supplement: Supplementary file 1 — Additional file 1: Supplementary Table 1. Absolute frequencies of study participants experiences with conducting, participating in and observing debriefings. [file 41077_2021_161_MOESM1_ESM.docx]

**Supplementary Table 1:** Absolute frequencies of study participants experiences with conducting, participating in and observing debriefings

| **Setting and activity** | Many (≥100) | Several (11-99) | Few (≤10) |
| --- | --- | --- | --- |
| Clinical debriefings: conducting | 2 | 7 | 27 |
| Clinical debriefings: participating | 1 | 6 | 27 |
| Simulation-based training debriefings: conducting | 8 | 4 | 25 |
| Simulation-based training debriefings: participating | 1 | 9 | 27 |
| Reflective debriefing practice (debriefing of debriefing): conducting | 2 | 6 | 28 |
| Reflective debriefing practice (debriefing of debriefing): participating | 2 | 5 | 27 |

Note. All participants provided quantifiable answers to the respective questions.
