# Supplementary material for: “A debriefer must be neutral” and other debriefing myths: a systemic inquiry-based qualitative study of taken-for-granted beliefs about clinical post-event debriefing
Source: Adv Simul (Lond). 2021 Mar 4;6:7. doi: 10.1186/s41077-021-00161-5 (PMC7931165; doi:10.1186/s41077-021-00161-5)
Supplement: Supplementary file 2 — Additional file 2: Supplementary Table 2. Characteristics of debriefings with respect to participants, place, duration, frequency, and organizational routines. [file 41077_2021_161_MOESM2_ESM.docx]

**Supplementary Table 2:** Characteristics of debriefings with respect to participants, place, duration, frequency, and organizational routines

| **Key theme** | **Representative Quote** | **%** |
| --- | --- | --- |
| ***Formal debriefing type*** | | |
| Various (e.g. team meetings, case reviews, shift changes, simulation-based training) | “[…] I’m commonly talking about recent situations and we also give feedback but we have hardly any structured debriefings.” | 39.3 |
| Following critical events | “[…] after a resuscitation, we discuss in a standardized manner with all people involved what went well and what went wrong […].” | 33.9 |
| None | “Not systematically.” | 14.3 |
| Intra-professional (nurses or physicians) | “[…] That was mostly with nurses talking among themselves […].” | 7.1 |
| Spontaneous | [“…] due to my own initiative or my colleagues’ initiative. We then met and someone structured the conversation […].” | 4.5 |
| ***How often*** | | |
| Not specified |  | 32.3 |
| Several times a year |  | 16.1 |
| Rarely |  | 12.9 |
| Daily |  | 12.9 |
| Variable |  | 6.5 |
| Several times a week |  | 26.5 |
| Once a week |  | 26.5 |
| Once a month |  | 26.5 |
| ***Duration*** | | |
| Not specified |  | 33.3 |
| $\leq60 min$ |  | 5.6 |
| $\leq45 min$ |  | 38.3 |
| $\leq30 min$ |  | 30.6 |
| $\leq15 min$ |  | 22.2 |
| ***Participants*** | | |
| All people involved | “For big debriefings (e.g. in the trauma bay), usually all people involved participate.” | 21.6 |
| Physicians and nurses | “[…] physicians and nurses are always participating […]” | 18.9 |
| Nurses | “[…] exclusively nurses […]” | 18.9 |
| Physicians | “Physician and physician, nurses are not participating.” | 16.2 |
| External debriefer | “[…] an external person listening […]” | 8.1 |
| [Interdisciplinary](https://www.dict.cc/englisch-deutsch/interdisciplinary.html) team members | “[…] especially with anaesthesia and trauma […]” | 8.1 |
| Own (professional) discipline | “All debriefings were conducted in anaesthesia teams.” | 5.4 |
| Not all people involved |  | 2.7 |
| ***Initiated by …*** |  |  |
| Attending physician | “[…] in the clinical setting, the attending physician is initiating it. He heard rumours and initiated a debriefing […].” | 25.0 |
| Most senior person | “Someone on a higher hierarchical level.” | 17.5 |
| Nurses | “[…] nurses have a certain feeling for initiating debriefings […].” | 17.5 |
| Physicians | “Mostly initiated by me (attending) but also by residents […].” | 15.0 |
| Another person | “[…] The one who feels called upon to do so.” | 12.5 |
| One discipline (e.g. trauma surgery, psychologists) | “Trauma surgeons are leading and also anaesthetists.” | 10.0 |
| Quality management | “[…] the quality management of the hospital demands it […], we have to show that we have talked about it […], it is a must […].” | 2.5 |
| ***Led by …*** | | |
| Attending physician | “[…] an attending is leading the debriefing […].” | 28.6 |
| Most senior person | “Mostly the person with the highest hierarchy level, mostly the attending physician or the nurse in charge.” | 25.0 |
| No one | “[…] without an instructor but it worked.” | 14.3 |
| The one who requests it | “The one who requests it because s/he noticed that something is wrong […].” | 10.7 |
| Trained and experienced staff | “Basically everyone with specific training.” | 10.7 |
| Psychologists | “[…] the one in the operating room was led by a psychologist.” | 7.1 |
| External trained debriefer | “External debriefer from other departments […] because they can ask naïve questions.” | 3.6 |
| ***Procedure*** | | |
| Specific structure | “Every participant first expressed her/his emotional reactions and talked about what went well and what went wrong. Based on these expressions, we chose and discussed two learning objectives and finished the debriefing by take-aways with respect to future performance.” | 77.3 |
| Little / no structure | “[…] if no one has a good structure, we are talking about the history of events and the most experienced person gives an evaluation […].” | 18.2 |
| Focus on technical aspects | “[…] focuses on technical aspects, enquiring and discussing knowledge […].” | 4.5 |
| ***Place*** |  |  |
| Private setting | “I try to find a separate and silent room […] without disruptions.” | 31.4 |
| Anywhere | “Where it happens to be, there are no predetermined rooms.” | 14.3 |
| Break room | “In the kitchen where we can eat and drink.” | 14.3 |
| Office | “In an office from the anaesthesia department.” | 11.4 |
| Trauma room | “In the trauma bay when we are waiting for the patients to be handed off. “ | 8.6 |
| Conference room | “After a child has died, we had to organize a conference room for the 25 participants of the debriefing.” | 8.6 |
| Operating room | “In situ in the operating room.” | 5.7 |
| Area for induction of anaesthesia | “In the area where we perform the induction of anaesthesia induction before we are moving to the operating room.” | 2.9 |
| Laboratory | “In our laboratory.” | 2.9 |
