# Supplementary material for: “A debriefer must be neutral” and other debriefing myths: a systemic inquiry-based qualitative study of taken-for-granted beliefs about clinical post-event debriefing
Source: Adv Simul (Lond). 2021 Mar 4;6:7. doi: 10.1186/s41077-021-00161-5 (PMC7931165; doi:10.1186/s41077-021-00161-5)
Supplement: Supplementary file 3 — Additional file 3: Supplementary Table 3. Beliefs about value of debriefing. [file 41077_2021_161_MOESM3_ESM.docx]

**Supplementary Table 3:** Beliefs about value of debriefing

| **Key themes** | **Representative Quote** | | **%** |
| --- | --- | --- | --- |
| ***On a scale from 1 (completely useless) to 10 (completely useful), where do you see the use of clinical debriefings?*** | | | |
| 9-10 |  | | 72.0 |
| 7-8 |  | | 21.6 |
| 5-6 |  | | 2.7 |
| 3-4 |  | | 2.7 |
| ***What should be different to support the use of debriefings?*** | | | |
| Culture change | “It should be accepted and an integral part of the daily business. Otherwise it will remain a ‘kaffeeklatsch’ […]” | | 42.1 |
| Available tool | “[…] we should do it more frequently and in a systematic manner with a tool […]” | | 31.6 |
| Improved logistics | “[…] due to shift work, it is problematic to bring all participants together.” | | 15.8 |
| More time | “[…] we do not have time to discuss different points in detail […].” | | 5.3 |
| Noticeable benefits | “Ensuring a transfer […] and noticeable benefits.” | | 5.3 |
| ***Who benefits the most from debriefings?*** | | | |
| Participants | “Everyone participating benefits even if it’s only ‘I knew it and I confirmed it’” […]. | | 41.8 |
| Junior / less experienced staff members | “Less experienced people benefit the most from debriefings […] maybe they learn how to handle different situations and hear how their colleagues managed it […]” | | 23.6 |
| Senior / more experienced staff members | “…those who have experience and don’t get any feedback [in the clinical setting].” | | 10.9 |
| People with a positive attitude | “Mostly people that are open minded and open to benefit from the debriefing […]. It depends on the acceptance and to see the whole point of it […].” | | 10.9 |
| Patients | “[…] learning to handle difficult situations also benefits patients.” | | 9.1 |
| Debriefer | “Those who facilitate it will benefit from it, they can develop personally, which is good.” | | 3.6 |
| ***If debriefings were conducted regularly and in the way that you deemed ideal, who would be the first to notice that the debriefings are valuable?*** | | | |
| Participants | | “I think at first the team members who have been debriefed will notice it […]” | 26.8 |
| Others (e.g., those who run debriefing; those who have been against debriefings; families of participants) | | “[…] maybe the families of the people who were in the debriefings because they will arrive at home more relaxed […]” | 24.4 |
| Staff | | “[…] dealing with each other during clinical work […]” | 19.5 |
| Junior / less experienced / less powerful staff members | | “I think that in hierarchical teams those who have the least power will be the first to realize how valuable that is […]” | 12.2 |
| Patients | | “The patients will soon notice it as well, I don’t think that they are the last. They are in their little cubbyhole and they extremely sensitive to the climate at the ER […] they notice how people talk to each other. They notice how coordination is going.“ | 9.8 |
| Everybody | | “Everybody shall notice it. […]” | 7.3 |
| ***What would they notice?*** | | | |
| Higher quality and fewer mistakes | | “[…] that fewer errors happen, working hours, that deficiencies are uncovered like working eight hours with neither eating nor drinking and that staff and patients will benefit from it […]” | 40.0 |
| Staff feels better / higher satisfaction / better climate | | “[…] I’m convinced that through debriefings working climate will improve […] “ | 28.9 |
| More open communication | | “[…] the culture, that one dares to address issues outside of debriefings or across the surgical cloth […]” | 20.0 |
| Better working conditions | | “[…] that there is less burnout or something like that.” | 11.1 |
| ***Who benefits the least from debriefings?*** | | | |
| Staff members with a negative attitude | “[…] the one who think it’s a waste of time and don’t value it.” | | 34.6 |
| Management | “[...] the people who define processes and who are far away from the frontline.” | | 15.4 |
| Absentee members & non-involved team members | “[…] I don’t believe that those who were not involved would benefit.” | | 15.4 |
| Senior / more experienced staff members | “I think the higher the position a participant in the debriefing has, the trickier it is for them.” | | 11.5 |
| Poor debriefing conduct | “It depends on the people doing the debriefings, on what people get from it.” | | 11.5 |
| Nurses | “The nurses usually have the fewest opportunities and the least power and authority to implement what they have learned in their daily work.” | | 7.7 |
|  |  | |  |
| Only positive feedback | “In those situations in which only positive feedback is given.” | | 3.9 |
| ***Who would be the last to notice the value of debriefings?*** | | | |
| Management | | “[…] high above in the hierarchy […] head of units […]” | 36.4 |
| Patients | | “[…] And at last the many thousands of patients, to whose safety we contribute, would notice it.” | 21.2 |
| Others (e.g., those who do not work with patients anymore; physicians) | | “Those superiors who don’t work clinically anymore, because they don’t realize when people are not well or when errors happen, they don’t realize it, except if something went really wrong.” | 21.2 |
| Those who do not like debriefings | | “[…] those who can’t be bothered with debriefings […]” | 12.1 |
| Those who did not participate in debriefings | | “[…] and at last those who did not participate […]” | 9.1 |
|  |  | |  |
| ***If one ‘saved’ the time spending in debriefings, what do you think would be lost?*** | | | |
| Learning | “The opportunity to learn from mistakes.” | | 38.5 |
| Communication & teamwork skills | “I think you would lose your ability to improve communication skills. | | 13.9 |
| Cultural change | “It changes the culture and you notice it every day.” | | 12.3 |
| Development of coping strategies & burnout prevention | “[…] they develop feelings of guilt and shame and these can prevent employees from wanting to work on this in similar cases.” | | 12.3 |
| Reflection | “[…] the really structured reflection, what was good and bad and what it means in everyday work.” | | 7.7 |
| Trust & confidence | “I think this binds people together and builds trust.” | | 6.2 |
| Reducing turnover | “[…] there is the risk that you lose an employee after a make-or-break/critical situation, if they blame themselves thinking that the outcome could have been different.” | | 4.6 |
| Positive work attitude | “You can see a positive atmosphere and the employees’ positive work attitude.” | | 4.6 |
| ***In your view, what is the difference between debriefing and normal conversation?*** | | | |
| Structure | “[…] if you approach it in a structured way and if you have guidelines you would like to talk about, you’ll reach the goal faster and in a more structured way.” | | 34.8 |
| Double-loop learning | “[…] if I want to find out why someone did something the way they actually did I it.” | | 18.8 |
| Goal-driven | “[...] but you are pursuing a specific goal, and this makes the difference to me.” | | 11.6 |
| Psychological safety | “[…] this is a protected area, we share views and no one will leak it. And people are asked to speak to each other with respect.” | | 11.6 |
| Facilitation | “[…] and the questions based on psychology, which I appreciate a lot, because you can ask them and still explicitly point out the mistake.”  “A debriefing […] is led.” | | 10.1 |
| Involvement | “The presence of everyone involved.” | | 7.3 |
| No emotions | “I assume that a debriefing […] explains with plain facts what the matter is, bluntly, with actual facts, objectively, without emotions.” | | 4.4 |
| Follows briefing | “The term debriefing says that there must have been a briefing before.” | | 1.5 |
