# Supplementary material for: “A debriefer must be neutral” and other debriefing myths: a systemic inquiry-based qualitative study of taken-for-granted beliefs about clinical post-event debriefing
Source: Adv Simul (Lond). 2021 Mar 4;6:7. doi: 10.1186/s41077-021-00161-5 (PMC7931165; doi:10.1186/s41077-021-00161-5)
Supplement: Supplementary file 4 — Additional file 4: Supplementary Table 4. Characteristics of a “good debriefer”. [file 41077_2021_161_MOESM4_ESM.docx]

**Supplementary Table 4:** Characteristics of a “good debriefer”

| **Key theme** | **Representative Quote** | **%** |
| --- | --- | --- |

| ***What characterizes a good debriefer?*** | | |
| --- | --- | --- |
| Respectability, standing & calmness | “They need to have a lot of staying power. Not giving in in situations that are emotionally burdensome and sticking to the topic.” | 24.6 |
| Skilled in facilitating conversations | “[… ] you need to be professionally skilled, regarding conversation technique; being able to keep the structure […]” | 21.5 |
| Empathy | “They need to know who in that situation might feel worst, who might feel good […]” | 13.9 |
| Being curious and listening | “That you are able to listen.” | 10.8 |
| Having meta-perspective | “[…] that you have the overview, are the participants participating, are they arguing, like monitoring how the conversation is going.” | 9.2 |
| Being neutral | “That s/he is neutral […]” | 6.2 |
| Being convinced of debriefing | “[…] I also think that you’ll notice if people are convinced of debriefing, and the participants will notice that, too; they’ll notice if somebody is just standing there and does it because it needs to be done […]” | 4.6 |
| Being self-reflective | “[…] If you can perceive and maintain your role” | 4.6 |
| Having medical knowledge | “[…] Indeed some medical insights […] that is important, the medical background knowledge [ …]” | 4.6 |
