# Supplementary material for: “A debriefer must be neutral” and other debriefing myths: a systemic inquiry-based qualitative study of taken-for-granted beliefs about clinical post-event debriefing
Source: Adv Simul (Lond). 2021 Mar 4;6:7. doi: 10.1186/s41077-021-00161-5 (PMC7931165; doi:10.1186/s41077-021-00161-5)
Supplement: Supplementary file 5 — Additional file 5: Supplementary Table 5. Beliefs about the learning environment of debriefings. [file 41077_2021_161_MOESM5_ESM.docx]

**Supplementary Table 5:** Beliefs about the learning environment of debriefings

| **Key theme** | **Representative Quote** | **%** |
| --- | --- | --- |
| ***In your view, should there be rules for debriefings?*** | | |
| Yes | “Especially during clinical work […] where it might be a less safe setting where it is more important that you do not expose one another or talk each other down […]” | 97 |
| Don’t know | “Rules in a legal sense or in general? I don’t know.” | 3 |
| ***Which rules?*** | | |
| Apply a structure | “I believe there needs to be a guide that is taught in courses because you need a structure to give you security […]” | 27.8 |
| Define time and duration | “Maybe a rough time frame to prevent it to be too long.” | 12.5 |
| Define time slots for everybody to speak | “[…] Time slots for speaking, all need to be involved, […]” | 11.1 |
| Provide psychological safety | “I think there should be rules for the debriefing that everybody feels safe to say something […]” | 9.7 |
| High regard and curiosity | “[…] that you remain appreciative that it is the truth of the participants that counts and not mine […]” | 9.7 |
| Define who may initiative debriefing and whether participation is optional or mandatory | “I think it would be good if there were rules for mandatory conduct after certain events […]” | 8.3 |
| Provide confidentiality | “[…] Las-Vegas-Principle […]” | 7 |
| Find quiet place | “[…] that you have the required quietness, the room […]” | 5.6 |
| Define facilitators’ role | “[…] that you are a coach and not an instructor” | 4.2 |
| Define goal and how to measure it | “[…]What are the take home messages, what is the goal? And, as I said, the results should be measurable.” | 4.2 |
| ***There is the assumption that ‘psychological safety’ is necessary to talk about problems or doubts. On a scale from 1 to 10, how realistic is it to establish this psychological safety in debriefings; 1=very unrealistic, 10=very realistic?*** | | |
| 7-8 |  | 43.6 |
| 5-6 |  | 28.1 |
| 9-10 |  | 18.8 |
| 3-4 |  | 9.4 |
| ***If one had achieved psychological safety, what had one done?*** | | |
| Stick to rules with respect to structure, transparency, setting, time, objectives | “By mentioning the rules explicitly at the beginning, the preview of expectations […]” | 25.4 |
| Provide confidentiality | "[...] a closed room with little influence from outside.“ | 15.9 |
| Get support from trained, neutral, authentic debriefers | “I think at the beginning support from independent people is required.” | 12.7 |
| Positive experiences | “[…] The more people talk about it and experience that nothing is going to happen to me.” | 11.1 |
| Superiors as good model | “I believe that if superiors set an example of approaching you during clinical work and saying ‚let’s do a debriefing because of whatever reason‘ […]” | 11.1 |
| Be respectful | “[…] I think respect is an important point, mutual. Interacting respectfully with one another […]” | 9.5 |
| Get introduction into debriefing | “[…] if people are familiar with it and trained” | 7.9 |
| Culture change | “It’s a matter of culture […]” | 6.4 |
| ***On a scale from 1 to 10, should there rather be unilateral feedback or thorough discussion in debriefings (1=feedback only, 10=discussion)?*** | | |
| 9-10 |  | 50 |
| 7-8 |  | 23.1 |
| 5-6 |  | 19.2 |
| 3-4 |  | 7.7 |
| ***In your view, how much patience do your colleagues have in debriefings to explore reasons for mistakes in detail (1=no patience at all, 10=much patience)?*** | | |
| 3-4 |  | 48 |
| 7-8 |  | 20 |
| 5-6 |  | 16 |
| 1-2 |  | 8 |
| 9-10 |  | 8 |
| ***In addition to no time and no patience, what might prevent people from exploring mistakes in debriefings?*** | | |
| Repercussions | “Well, the concern of legal consequence if somebody finds out that it would have been a preventable mistake; we would be sued. And the fear of having one’s own competence questioned.” | 64.3 |
| Interpersonal and cultural issues | “Well it is, I think, the culture that speaks against it, but it is getting better […]” | 25 |
| Feeling coerced | “A very high degree of feeling very uncomfortable, like on black ice, the feeling of not being able to handle things.” | 10.7 |
| ***With which feeling should participants leave at the end of a debriefing?*** | | |
| Good / content | “Content, I think […]” | 41.9 |
| Having learned something | “[…] having learned something and with insights […]” | 35.5 |
| Motivated | “feeling good and motivated” | 9.7 |
| Being heard | “[…] being understood […]” | 6.5 |
| Ö- | “[…] the feeling of having been able to leave a part of burden with the team and it’s not on my back anymore. That’s important.“ | 6.5 |
| ***If somebody would debrief you and your team, what must that person not do to you?*** | | |
| Blame | “Affront me, I think. It is not about blaming somebody but to focus on the problem and how everybody contributed; it’s not about pointing fingers.” | 35.3 |
| Attack | “To corner me in a way that I feel forced to say something.” | 23.5 |
| Humiliate | “[…] exposing me or a member of the team. ‘Why did you this—this is totally wrong.’ […]“ | 20.6 |
| Lecture | “Personally, I feel the condescending or the patronizing is the worst.” | 20.6 |
